# Learning benefits of live surgery and semi-live surgery in urology—informing the debate with results from the International Meeting of Reconstructive Urology (IMORU) VIII

**DOI:** 10.1007/s00345-020-03506-3

**Published:** 2020-11-02

**Authors:** Victor M. Schuettfort, Tim A. Ludwig, Phillip Marks, Malte W. Vetterlein, Valentin Maurer, Constantin Fuehner, Florian Janisch, Armin Soave, Michael Rink, Silke Riechardt, Oliver Engel, Margit Fisch, Roland Dahlem, Christian P. Meyer

**Affiliations:** Department of Urology, University Medical Center Hamburg-Eppendorf, Hamburg, Germany

**Keywords:** Live surgery events, Live surgery demonstrations, Semi-live surgery, Surgical education, IMORU, Reconstructive urology

## Abstract

**Purpose:**

To analyze the perceived learning opportunities of participants of the International Meeting on Reconstructive Urology (IMORU) VIII for both live surgery demonstrations (LSD) and semi-live surgery demonstrations (SLSD). Safety and educational efficacy of LSD and SLSD at live surgery events (LSE) have been debated extensively, however, objective data comparing learning benefits are missing.

**Methods:**

We conducted a detailed survey, which employed the Kirkpatrick model, a well-established assessment method of training models, to investigate participants preferences as well as the learning benefit of LSE. Furthermore, we employed an audience response system and the Objective Structured Assessment of Technical Skills (OSATS), a well-established assessment method of surgery skills, to let our participants rate the perceived learning opportunity of LSD and SLSD.

**Results:**

Of 229 participants at the IMORU VIII, 39.7% returned our questionnaires. 90% stated that they prefer LSD. On all levels of Kirkpatrick’s training evaluation model, the IMORU received high ratings, suggesting a high learning benefit. For the assessment of OSATS, a total of 23 surgical cases were evaluable. For all six utilized items, LSD scored significantly better ratings than SLSD.

**Conclusion:**

Our study suggests that there is still a rationale for LSD, as participants attributed a statistically significant higher learning benefit to LSD over SLDS. Evaluation of the survey showed that for LSE such as the IMORU VIII, a high learning benefit can be expected. Considering that most of our participants are active surgeons with high caseloads, their opinion on the educational value of LSE is of high relevance.

## Introduction

For decades, live surgery demonstrations (LSDs) have been accepted as an appropriate tool for surgical education. However, concerns about patient safety have led to an ongoing debate about the values of LSDs, with some surgical societies eventually banning LSDs altogether [1–7]. Semi-live surgery demonstrations (SLSDs), e.g., the moderated presentation of pre-recorded videos, have been proposed as a preferable alternative [8, 9]. While the debate on the pros and cons of LSDs and SLSDs continues, there is surprisingly little objective data to inform the discussion and learning benefits of either modality.

The *International Meeting on Reconstructive Urology* (IMORU) is a triennial meeting of reconstructive urologists, hosted by the Department of Urology of the University Medical Center Hamburg-Eppendorf since 2010. Among various presentations and exhibitions, it features LSDs from multiple on-campus operating theatres, as well as SLSDs and live broadcasted surgeries. The procedures are performed by internationally renowned experts, covering the entire spectrum of reconstructive urology. Recently, we were able to demonstrate acceptable and comparable complication rates during IMORU V and VI [10].

Our objective was to investigate the current value of surgical education meetings (SEMs) in urology and to compare the learning benefit of LSDs and SLSDs. We therefore analyzed the preferences and the perceived learning opportunity of our participants at the IMORU VIII for both LSDs and SLSDs using an audience response system and a detailed survey.

## Methods

The IMORU VIII took place in Hamburg between March 11 and 13, 2019. In the plenary hall of the congress, participants could view all streamed LSDs and SLSDs and follow the moderation. A total of 33 procedures performed by 22 surgeons were demonstrated, of which nine were SLSDs including five minimally invasive procedures. Two procedures were live broadcasted from abroad. The demonstrated procedures covered the entire spectrum of reconstructive urology (e.g., urethral and ureteral reconstruction, implantation of male slings, artificial urinary sphincters and penile prostheses, corporoplasties, and gender reassignment surgery). All LSDs were performed at the University Medical Center Hamburg-Eppendorf. SLSDs generally consisted of a moderated video screening by the surgeon of procedures recorded at their home institution. All participating surgeons were internationally renowned experts in the field of reconstructive urology, with experience in LSDs. European Association of Urology (EAU) guidelines on conducting live surgery events were followed [11]. All sponsoring companies of the IMORU VIII had no influence on patient treatment, the meeting itself or this study.

### Survey evaluation

At the final day of IMORU VIII, all participants were handed out a printed version of a non-validated, anonymous, and standardized multiple-choice questionnaire with default reply options. The survey was reviewed multiple times and finalized by the congress organizers in order to guarantee comprehensibility. The first part of the survey consisted of questions on participant’s information. Furthermore, we asked a specific set of questions concerning the learning effect of both LSDs and SLSDs for a comparison of participant’s preference.

The second part of the survey aimed at investigating the attributed learning benefit of SEMs like the IMORU. To do so, we used an adapted version of the Kirkpatrick model [12]. This model allows analyzing behavioral changes due to a learning activity. It is based on four different levels, which focus on the participant’s reaction (level one: perceptions and satisfaction), learning (level two: changes in skills or knowledge), behavior (level three: post-learning transfer in the practice setting), and results (level four: future impact on outcome) [12]. The model has been introduced in the sixties, and it is still frequently used to evaluate and review learning activities, as it has the ability to simplify the complex process of evaluation in training programs [13–16].

In our adaption of the Kirkpatrick model, we measured the participants’ reactions to the SEM (level one) by asking the attendees about their general perception of the IMORU and its relevance for clinical practice. Evaluation of learning experience (level two) was performed by reviewing the perceived learning opportunities of the meeting with respect to indications, alternatives, key steps, and pitfalls in reconstructive urology. For evaluation of post-learning behavior (level three), only participants of at least one previous IMORU were asked about the influence of the meeting on their clinical practice and surgical capabilities. For evaluation of the estimated results of participation (level four), we asked all participants to rate their expected influence of the IMORU on their surgical outcome and complication rates. All items were to be rated using a Likert scale as displayed in Table 1.

**Table 1:** Extended participant information and assessment of participant’s perception of the IMORU VIII using the Kirkpatrick model *(all ratings using a Likert scale 1–5/good to bad)*

Extended participant information	*N*	Mean	SD	Min	Max
Number of years performing reconstructive urology	82	12.63	8.64	1	35
Number of performed reconstructive surgeries in a year	79	83.68	58.94	5	300
Number of performed urethroplasties per year	73	53.02	52.97	3	250
Number of memberships in professional societies	81	1.98	1.27	1	6
Number of live surgery events attended	64	4.68	3.91	1	20
Number of IMORUs attended *(for those who have previously attended an IMORU)*	42	3.40	2.11	1	8

Assessment of participant’s perception of the IMORU VIII using the Kirkpatrick model	*N*	Mean (*Likert scale 1–5)*	SD	Min	Max
Assessment of participant reaction (Level 1)					
How would you rate the overall surgical performance	92	1.23	0.44	1.00	3.00
Did you find the surgeries performed relevant for your own practice	92	1.24	0.56	1.00	4.00
Did you find tips and comments relevant	91	1.19	0.49	1.00	4.00
Did you find the IMORU a beneficial meeting	91	1.12	0.36	1.00	3.00
How would you rank your learning experience	91	1.36	0.51	1.00	3.00
Assessment of participant learning (Level 2)					
Do you feel you learned about the indications for reconstructive surgery	91	1.69	0.81	1.00	5.00
Do you feel you learned about alternative surgery options	91	1.48	0.67	1.00	4.00
Do you feel you learned about pitfalls in reconstructive urology	91	1.55	0.81	1.00	5.00
Do you feel you learned you have been reminded of key steps of the demonstrated surgeries?	91	1.35	0.67	1.00	4.00
Assessment of participant behavior (Level 3) *only for participants that previously attended an IMORU*					
Do you use knowledge or skills acquired at the IMORU	45	1.36	0.57	1.00	3.00
Did the way you perform reconstructive surgeries change after visiting the IMORU	44	1.8	0.67	1.00	4.00
Did your surgical armamentarium expand after visiting the IMORU	44	1.91	0.83	1.00	4.00
Did your outcome or success rate of your reconstructive surgery change after visiting the IMORU	44	1.90	0.74	1.00	3.00
Did your complication rate of your reconstructive surgery change after visiting the IMORU	44	1.98	0.73	1.00	3.00
Do you feel better at handling complications during reconstructive surgery after visiting the IMORU	45	2.13	1.24	1.00	5.00
Assessment of participant outcome (Level 4)					
Rate the expected impact of the IMORU meeting on your surgical performance	89	1.53	0.57	1.00	3.00
Rate the expected impact of IMORU on your complication rate	84	1.83	0.74	1.00	3.00
Rate the expected Impact of IMORU on your surgical outcome	86	1.73	0.58	1.00	3.00

*N* number of respondents, *SD* standard deviation

### Comparison of LSEs and SLSDs using Objective Structured Assessment of Technical Skill (OSATS)

For nearly all procedures performed at the IMORU VIII, participants were able to rate the perceived learning opportunity of the intervention. For this, an anonymous audience response system (H-ITT®) and the corresponding clickers (H-ITT iCue RF®) and software (H-ITT CRS®) were used. Clickers were handed out to all present attendees. Using a Likert scale, the participants were asked to rate how well they were able to observe the surgeons with respect to tissue, timing, motions, instrument handling as well as key steps and pitfalls of each demonstrated procedure. The participants were also asked to rate the overall surgical presentation and the learning benefit of the demonstrated procedure. The questions employed are based on the OSATS. This is one of the first described methods for the assessment of surgical skills, which was thoroughly validated and can be considered the current gold standard for surgical skills evaluation [17–19]. The OSATS system originally involved trainees rotating through various stations performing different surgical task, while a qualified senior surgeon assessed the performance using a Likert scale [20]. The complete set of OSATS items was not used, as participants perception of some features is very unlikely to differ between LSDs and SLSDs. Therefore, the originally included items concerning the surgeon’s knowledge of instruments, use of assistance, flow of operation, forward planning, and knowledge of the specific procedure were excluded from our adapted questionnaire. It is important to recognize that the participants were explicitly not asked how well the surgeon performed, but rather how well the viewer was able to observe and apprehend the different aspects of the surgery, turning the observer’s rating into a surrogate of the perceived learning opportunity. As the questions were the same for both LSDs and SLSDs, we generated a comparable question set, with which we are able to compare the learning benefit of both teaching modalities. Due to organizational and time reasons, not every surgical demonstration could be evaluated.

### Statistical analysis

For analysis of the OSATS scoring, statistical analyses featured a descriptive analysis and a two-sided independent t test for the surgical approach (open vs. minimally invasive surgery) and the mode of demonstration (LSD vs. SLSD). Further variables such as the type of surgery and the surgeon were excluded from the analyses, given that due to the high number of categories, any statistical analysis would have been underpowered. Evaluation of the survey focused on percentages, means, and standard deviation (SD) for categorical or continuous variables. All statistical analyses were performed using SPSS 20.0 (SPSS Inc.) with significance level set at *P* < 0.05.

## Results

### Baseline Characteristics of the Participants

A total of 229 attendees from 42 countries participated at the IMORU VIII. A total of 91 questionnaires were returned, corresponding to a response rate of 40%. The vast majority (*n* = 86, 94%) were actively performing reconstructive urologists. Most of them report long experience (mean: 13 years, SD: 8.6) and a high case load of in reconstructive urology (mean: 84 surgeries per year, SD: 68). The majority reported experience in performing urethroplasties (*n* = 75, 82%) and a high case load of performed urethroplasties (mean: 53 per year SD: 53). The participants were quite frequently members of at least one professional society (*n* = 82; 89%) and regularly attend SEMs (*n* = 72, 78%). In total, 53% (*n* = 49) had attended an IMORU before. All attendees stated that they would attend IMORU again (*n* = 91, 100%). Further participant information is presented in Table 1.

### Survey—Comparison between LSDs and SLSDs

Most participants stated that they prefer LSDs (*n* = 81/90, 90%) over SLSDs. For the majority of responders, LSDs are an appropriate tool for surgical education (*n* = 86/91, 94%). The majority felt that there are little or no ethical concerns with conducting a SEM that features LSDs (*n* = 66/91 73%), and only few would argue that SLSDs are ethically less concerning than LSDs (*n* = 29/87 33%). The rating of the educational value of LSDs and SLSDs (rated 1–5) showed a significant better average score for LSDs (mean: 1.4, SD 0.053) in comparison with SLSD (1.8; SD 0.062; *n* = 90, mean difference 0.37, SD 0.07, 95% CI 0.20–0.54; *p* < 0.001).

### Survey—Participants’ learning evaluation—Kirkpatrick’s model

On all four levels of Kirkpatrick’s model, the participants perceived a high learning benefit at the IMORU VIII (Table 1). The participants showed very positive reaction to the meeting (level one). Assessment of the second level showed that the participants also perceived a high learning benefit. Participants who previously attended an IMORU stated they have noted an improvement in their surgical outcome and skills after the attendance (level three). Furthermore, most participants expect a positive influence of the IMORU VIII on their surgical outcome (level four).

### OSATS

For the evaluation of OSATS, a total of 23 procedures by 17 different surgeons were eligible for evaluation (18 LSDs, 5 SLSDs). Three evaluable procedures were performed with a laparoscopic or robot-assisted approach. On average, each procedure received a total of 175 different ratings on all six questions (SD: 80). There was no significant difference between the average number of ratings for LSDs and SLSDs (175 vs. 175; *p* = 0.84). For all six questions of the OSATS questionnaire, as well as the mean overall score, LSDs scored significantly better than SLSDs (Table 2). Surgical approach did not significantly affect mean rating for all six questions (all *p* > 0.05).

**Table 2:** OSATS scores of live surgery demonstrations and semi-live surgeries demonstration at the IMORU VIII *(all ratings using a Likert scale 1–5/good to bad)*

	*N*	Mean	SD	Mean difference	95% CI of the Mean difference		*p*
					Lower	Upper	
Question 1: How well were you able to assess and observe the surgeons respect for tissue?							
LSD	853	1.75	1.11	− 0.23	− 0.39	− 0.06	0.008
SLSD	224	1.98	1.19				
Overall	1077	1.8	1.13				
Question 2: How well were you able to assess and observe the surgeons timing and motions?							
LSD	586	1.85	1.13	− 0.25	− 0.44	− 0.06	0.012
SLSD	178	2.1	1.20				
Overall	764	1.91	1.15				
Question 3: How well were you able to assess and observe the surgeons instrument handling?							
LSD	565	1.87	1.08	− 0.22	− 0.40	− 0.03	0.024
SLSD	167	2.08	1.11				
Overall	732	1.92	1.09				
Question 4: How well were you able to assess and observe key steps and pit falls of the performed surgery?							
LSD	509	1.91	1.15	− 0.24	− 0.45	− 0.03	0.028
SLSD	141	2.15	1.13				
Overall	650	1.96	1.15				
Question 5: Please rate the overall surgical presentation							
LSD	366	1.86	1.12	− 0.26	− 0.52	− 0.01	0.039
SLSD	100	2.12	1.17				
Overall	466	1.91	1.14				
Question 6: Please rate the learning effect of the performed surgery							
LSD	261	1.90	1.16	− 0.38	− 0.68	− 0.09	0.012
SLSD	77	2.29	1.15				
Overall	338	1.99	1.17				
Average Overall Score							
LSD	1032	1.8	1.05	− 0.20	− 0.34	− 0.07	0.03
SLSD	307	2.0	1.08				
Overall	1339	1.85	1.06				

*N* Overall number of responses, *LSD* live surgery demonstration, *SLSD* semi-live surgery demonstration, *CI* Confidence Interval, *p* *p* value

## Discussion

To the best of our knowledge, this is the first study that objectively compares LSDs and SLSDs in urologic reconstructive surgery. Using the OSATS score, our participants attributed a statistically significant higher learning benefit to LSDs with respect to multiple different attributes. These results are in concordance with our performed survey, in which we found that participants credit LSDs with a significant higher learning benefit. Evaluation of a Kirkpatrick model showed that for SEMs such as the IMORU, a high learning benefit can be expected, given that attendees attributed very positive ratings on all four levels. Participants who previously visited an IMORU reported a positive influence on their daily practice and surgical performance. Furthermore, most participants estimate a positive influence on their future outcomes. Considering that most of our participants are active surgeons with high caseloads, their opinion on the educational value of SEMs such as the IMORU is of high relevance.

So far, the comparison of LSDs and SLSDs is only based on data from surveys. Finch et al. questioned 165 participants at the UK section meeting of the Société Internationale d'Urologie (SIU) in 2013. In their survey, responders felt that the educational value of LSDs and SLSDs was similar, while attributing a significant higher patient safety benefit to SLSDs [9]. In the survey by Phan et al. conducted at the World Congress of Endourology in 2015, participants again perceived little difference in the learning benefit of both LSDs and SLSDs, leading the authors to the conclusion that SLSDs are non-inferior as an educational tool [8]. A recently performed survey by Legemate et al. conducted at the 2017 Challenges in Endourology congress reiterated that attendees attribute LSEs and SLSDs the same learning experience, while a substantial percentage of surgeons who had performed LSDs stated patient safety may be jeopardized. Nevertheless, one-third of the responders would attend fewer SEMs if SLSDs were to replace LSDs [21]. This finding suggests that despite all technological progress and benefits of SLSDs, LSDs remain a popular tool for surgical education.

Contrary to these previous studies, our data suggest that participants of the IMORU VIII prefer LSDs over SLSDs. One of the reasons why our participants attributed LSDs a higher learning benefit might be the proposed bias of pre-selection in SLSDs [3]. LSDs potentially offer a more genuine and natural surgical demonstration, leaving little to no room for adjustments in a real-world surgical environment. Furthermore, most of our responders stated that they feel there is little ethical dilemma while conducting LSDs, offering a different perspective on one of the major concerns of LSDs. Indeed, multiple studies reported no higher complication rate following LSDs despite strong theoretical concerns for patient safety [10, 22–26].

As of now, due to demonstrated patient safety and learning benefit, there seems to be a rationale to continue LSDs. However, our results should not be interpreted as suggesting that LSDs are superior to SLSDs. The numerous ethical dilemmas with LSDs remain a challenging subject [27]. Multiple surveys found that the participants of SEMs would not subject themselves or a family member to LSDs [3, 9, 28]. SLSDs carry significant logistical advantages over LSDs, reducing the risk of time wasting and thus maximizing efficiency of educational meetings. SLSD is presumably more cost-effective and less labor-intensive than extensive LSDs with multiple teams, live transmission, etc. More fundamentally, SLSDs allow the surgeon to focus on the surgery and cater the presentation more toward the audience. Stress for the surgeon is reduced as the surgery takes place in a known and familiar environment. Furthermore, pre-recorded videos offer the advantage to be altered in a way that the learning value can be increased. This carries no real disadvantages except the previously mentioned pre-selection bias. The demonstration can be paused for a detailed explanation, while key steps can be repeated or simplified in an abstract manner through pictures or animations. However, the presentation of SLSDs needs just as much care as the demonstration of LSDs. The teaching skills of the surgeon, the ability to simplify hard to grasp concepts, are equally important regardless of the medium.

A potential limitation is the response rate of only 40%. Furthermore, there is a potential selection bias, as those who attend SEMs are very likely to be positively inclined toward LSDs. Also, the assessment of a learning benefit is a difficult due to the lack of validated tools. For the unique scenario of IMORU VIII, we needed to adapt the OSATS questionnaire and the Kirkpatrick model and were not able to collect pre/post-test data. Our results might also be influenced by other unaccountable factors like the degree of difficulty of the surgery or screening time. Furthermore, the results may only display a role for LSDs in reconstructive surgery, where intraoperative problem solving is key and demands flexible and experienced surgical decisions in a highly specialized environment. As there is only little data on this highly relevant topic, further studies are needed. Evaluation of pre- and post-test and adjustment for further variables could allow for an in-depth comparison between LSDs and SLSDs.

## Conclusion

In conclusion, our results suggest that there is currently still a role for LSDs in surgical education. However, as SLSDs carry several fundamental and logistical advantages, further technological and methodological refinements may give it the edge over LSDs in the future.

## Abbreviations

IMORU: International Meeting on Reconstructive Urology
LSD: Live surgery demonstration
SLSD: Semi-live surgery demonstration
SEM: Surgical education meeting
OSATS: Objective Structured Assessment of Technical Skills
EAU: European Association of Urology
SD: Standard deviation
